# Diet Therapeutics Interventions for Obesity: A Systematic Review and Network Meta-Analysis

**DOI:** 10.34172/jrhs.2021.63

**Published:** 2021-09-19

**Authors:** Mina Morsali, Jalal Poorolajal, Fatemeh Shahbazi, Aliasghar Vahidinia, Amin Doosti-Irani

**Affiliations:** ^1^Department of Epidemiology, School of Public Health, Hamadan University of Medical Sciences, Hamadan, Iran; ^2^Modeling of Noncommunicable Diseases Research Center, Hamadan University of Medical Sciences, Hamadan, Iran; ^3^Department of Biochemistry and Nutrition, School of Medicine, Hamadan University of Medical Sciences, Hamadan, Iran; ^4^Research Center for Health Sciences, Hamadan University of Medical Sciences, Hamadan, Iran

**Keywords:** Diet therapy, Network meta-analysis, Obesity

## Abstract

**Background:** Up to now, different diet therapeutics interventions have been introduced for the treatment of obesity. The present study aimed to compare the diet therapeutics interventions for obesity simultaneously.

**Study design:** Systematic review and network meta-analysis

**Methods:** The major international databases, including Medline (via PubMed), Web of Science, Scopus, Cochrane Library, and Embase, were searched using a predesigned search strategy. Randomized controlled trials (RCTs) that had compared the diet therapy interventions were included. The mean difference with a 95% confidence interval was used to summarize the effect size in the network meta-analysis. The frequentist approach was used for data analysis.

**Results:** In total, 36 RCTs out of 9335 retrieved references met the inclusion criteria in this review. The included RCTs formed nine independent networks. Based on the results, Hypocaloricdiet+Monoselect Camellia (MonCam, P=0.99), energy restriction, behavior modification+exercise (LED) (P=0.99), sweetener at 20% of total calories (HFCS20)+Ex (P=0.67), catechin-richgreentea(650)+inulin (P=0.68), very low calorie diet (VLCD) (P=1.00), normal protein diet+resistance exercise (NPD+RT) (P=0.80), low-calorie diets+exercise (Hyc+Ex) (P=0.85), high-soy-protein low-fat diet (SD) (P=0.75), calorie restriction+behavioral weight loss (Hyc+BWL) (P=0.99) were the better treatments for weight loss in the networks one to nine, respectively.

**Conclusion:** Based on the results of network meta-analysis, it seems that Hypocaloricdiet+MonCam, LED, HFCS20+Ex, catechin-rich green tea +inulin, VLCD, NPD+RT, Hyc+Ex, SD, Hyc+BWL, are the better treatments for weight loss in patients with overweight and obesity.

## Introduction

Obesity, as a major public health problem^1^, is a medical condition in which excess fatty tissue is accumulated in a person's body ^2^. Obesity has major complications, such as reduced longevity and quality of life for patients. The most common measure for the diagnosis of obesity is the use of body mass index (BMI). According to World Health Organization (WHO), BMIs over 25 and 30 are considered overweight and obese, respectively. Overweight and obesity in the US and other industrialized countries represent a significant and growing health problem^2^. Each year, overweight and obesity lead to more than four million deaths globally. In 2016, the global prevalence of overweight and obesity among adults was 38% and 13%, respectively^1^. According to WHO, in 2020, the global prevalence of obesity varied from 2.1% in Vietnam to 36.2% in the US^3^.

Obesity is a major risk factor for many chronic diseases, such as high blood pressure, hyperlipidemia, cardiovascular diseases, type 2 diabetes, and cancer^4,5^. Obesity is a multifactorial condition, and its risk factors vary based on age. The common risk factors of obesity in adults are sedentary behaviors, use of diet with high fats and carbohydrates, and stress. Sedentary behaviors, parental obesity, and limited access to fruits and vegetables are major risk factors in adolescents. In infants, high maternal BMI, low birth weight, early termination of breastfeeding, and maternal diabetes are the major risk factors of childhood obesity ^6^. Modest weight loss of 5-10% of body weight significantly improves obesity-related chronic diseases^7^. It has been demonstrated that low-calorie diet interventions help in both short- and long-term weight reduction in individuals who are overweight or obese ^8^.

Until now, various diet therapeutic interventions for the treatment of obesity have been introduced ^9-14^. The role of diet composition in weight loss has been investigated extensively in randomized controlled trials (RCTs); however, there are controversies in their results^15^. In addition, no RCT could be found in which all the available diet therapeutics were compared simultaneously as the classic meta-analysis can compare only two treatments. Hence, it seems the published RCTs and classic meta-analyses cannot provide sufficient evidence regarding the simultaneous comparison of all available diet treatments.

The simultaneous comparison of these interventions offers useful information about the effectiveness of interventions for patients and clinicians. Network meta-analysis (NMA) is a valuable tool for the simultaneous comparison of more than two treatments. An NMA can compare two treatments, even when they have not been compared directly in any RCT. In addition, NMA allows investigators to rank treatments in terms of effectiveness in the network of treatments^16^. Therefore, this NMA aimed to simultaneously compare the available diet therapy interventions for weight loss in obese patients.

## Methods

This systematic review and NMA was reported based on the PRISMA-NMA statement^17^. This paper is a part of a comprehensive systematic review that compares all treatment options for the management of obesity. The proposal of this study was approved by the Ethics Committee of Hamadan University of Medical Sciences, Hamadan, Iran (IR.UMSHA.REC.1398.833).

###  Search strategy

The major international databases, including Medline, Web of Science, Scopus, Cochrane Library, and Embase, were searched until January 2020. We developed a search strategy to find the published RCTs that had evaluated the diet therapy interventions for the treatment of obesity (Supplementary Table 1). In addition, it should be mentioned that the reference lists of the included RCTs were scanned.

###  Selection criteria 

*Type of studies:* In this systematic review and NMA, the published RCTs of therapeutic interventions for the treatment of obesity were included, regardless of the study site, time, and language of publication. Other study designs, such as case reports, case series, and retrospective and prospective cohort studies, were excluded.

*Type of population studied:* In this NMA, the study population consisted of patients with obesity or overweight who participated in RCTs that evaluated the diet therapies for weight loss. The RCTs that assessed the therapeutic intervention on patients with chronic diseases were excluded.

*Data extraction:* Two reviewers (M.M. and F.Sh.) were responsible for screening the retrieved references. All the retrieved preliminary studies were imported into EndNote software version X8, and the duplicate studies were removed in the first step. In the following step, the remaining studies were screened independently based on the title and abstract by the two aforementioned reviewers. Any disagreement between the reviewers was resolved by discussion and the judgment of a third reviewer (A.D.I.). Afterward, the full text of the selected RCTs was reviewed based on the eligibility criteria.

 The following categories of data were extracted: 1) data regarding the characteristics of RCTs, including the name of the first author, year of publication, location of study, duration of follow-up, the approach for data-analysis (intention-to-treat or per-protocol), study population, and sample size; 2) data regarding the interventions, including the exact type of diet interventions in each arm of RCTs; 3) the potential effect modifiers, including baseline BMI, gender, and mean age of participants; 4) the outcome, including the baseline mean value and standard deviation (SD) of the weight in participants, the mean and SD of the weight of the participants after the follow-up, the mean difference (MD) and SD of weight loss before and after of intervention in each arm of RCTs, the MD with SD or 95% confidence interval (CI) of weight loss between arms of RCTs.

It must be noted that the unit of weight loss in this study was kilograms. If the mean and SD of weight were reported in pounds, they were converted into kilograms. In the case of studies that instead of the mean weight, the median of weight and instead of the SD, the first and third quarters were reported, the mean and SD were calculated using the following formulas (1 and 2)^18^:


$$\begin{array}{l} \bar{X}=\frac{q_{1}+m+q_{3}}{3}\\ SD=\frac{q_{3-}q_{1}}{3} \end{array}$$

Here *q*_1_ and *q*_2_ are first and third quartiles, respectively, and *m* is median. Some of the included RCTs did not report the MD and SD for weights before and after the interventions, while the before and after weights and their SDs were reported in each arm. Therefore, the MDs were calculated by subtracting the post-intervention weight from the pre-intervention weight. Moreover, the following formula was used to calculate SD for the MD. In this formula, the correlation coefficient for the mean of weight before and after of intervention was considered 0.5^19^.


$$SD_{change}=\sqrt{SD_{baseline}^{2}+SD_{post}^{2}-\left(2\times corr\times SD_{baseline}\times SD_{post}\right)}$$

In studies that the 95% CI has been reported for MD, the SD was calculated using the following formula ^19^:


$$SD=\sqrt{N}\times \frac{upper\;\lim it-lower\;\lim it}{3.92}$$

###  Risk of bias assessment

The Cochran tool was used for the risk of bias assessment^20^. For this purpose, four items of this tool were selected, including random sequence generation, allocation concealment, blinding of outcome assessment, and incomplete outcome data. The included RCTs were low, intermediate, or high risk of bias if all mentioned items were met, if one item was not met and if more than one item were not met, respectively.

 Similar treatment interventions in the included studies were merged. In RCTs that the control group received no intervention, no exercise, and usual care; hence, the control group was considered the usual care group. Exercise interventions, without severity and duration, such as walking, boating, physical activity, and aerobic exercise, were considered exercise (Ex). Hypocaloric diet (Hyc) interventions, such as calorie restriction and low-calorie diet, were considered Hyc.

###  Similarity and consistency assumptions

The similarity assumption was evaluated in terms of clinical and epidemiological effect modifiers. The heterogeneity was assessed in the two-by-two comparisons in pairwise comparisons and the networks of interventions. The chi-squared test was used to check heterogeneity and I^2^ statistics quantified it. The loop-specific and design-by-treatment interaction approaches were used to assess the consistency assumption^21,22^.

###  Data analysis

The treatments in each network were presented visually by network plot^23^. Based on the change score analysis, the MD was used to summarize the treatment effects in the NMA. The results of NMA were reported with a 95% CI.

Treatments in each network were ranked using the p-value. The p-value is between zero and one, and the higher values indicate the better treatment. The p-value for treatment is calculated using the one-sided p-value of rejecting the null hypothesis (Pj). In a network, the p-value for each treatment is the mean of all 1-P[j] ^24^. Statistical analysis was conducted using R software version 4.0.0 (2020-04-24). The R package netmeta was used for NMA. The Review Manager software (version 5.4) was used for the risk of bias assessment^25^.

## Results

**Figure 1:**
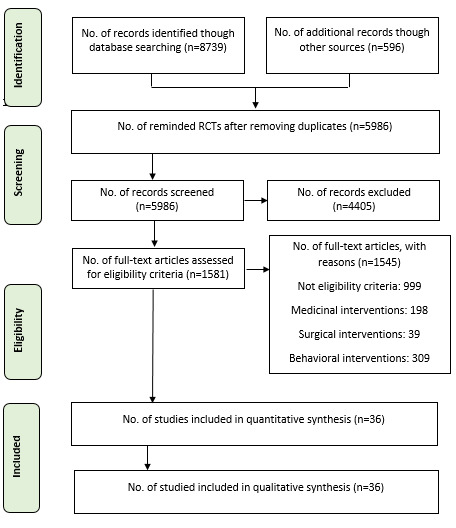
A flow chart showing the stages of retrieving articles and assessing the eligibility criteria for network meta-analysis of diet thereputics intervention for treatment of obesity

Overall, 601 RCTs out of 9335 retrieved references met the eligibility criteria for our comprehensive systematic review. Among these studies, 36 RCTs were related to diet therapy interventions for the management of obesity (Figure 1). The characteristics of included RCTs were presented in supplementary Table 2. The similarity assumption in terms of clinical and epidemiologic features was met for all the included RCTs. The included RCTs involved 36 RCTs with 68 treatments and 59 pairwise comparisons. Results of risk of bias assessment are shown in supplementary Figure 1. The treatments formed nine sub-networks with more than two treatments. It is noteworthy that 28 treatments in 14 RCTs were not connected to any network, and their results were reported in supplementary Table 2.

**Figure 2:**
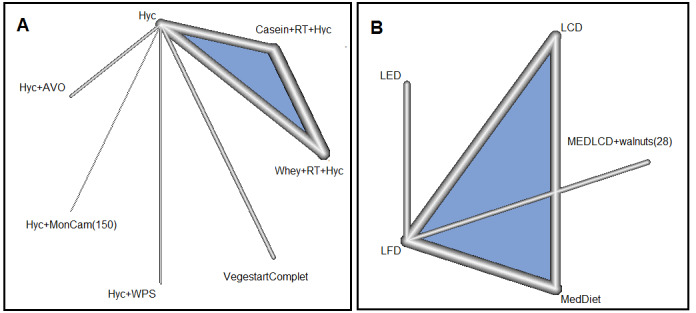
Networks one (A) and two (B) for diet therapeutics interventions of obesity.

**Figure 3:**
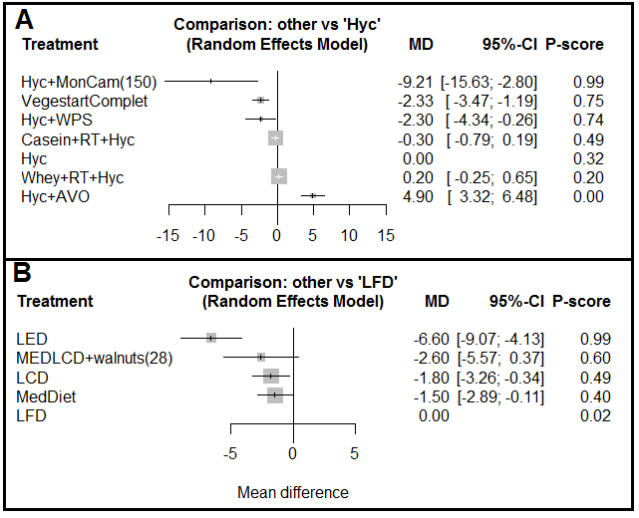
Forest plot for comparison of all treatments in network one with Hyc (A) and all treatments in network 2 with LFD (B) with related p-value for ranking of each treatment.

***Network 1***: This network involved five RCTs with seven treatments and seven two by two comparisons (Figure 2 A). The value for I^2^ was zero, and the network was consistent. The Hyc+Monoselect Camellia (MonCam) treatment (150) (P=0.99, MD=-9.21 [-15.63, -2.80]), VegestartComplet [P=0.75, MD=-2.33 (- 3.47, -1.19)], and Hyc+VPS (Hypocaloric diet+supplementation with whey protein) [P=0.74, MD=-2.30 (-4.34, -0.26)] versus Hyc alone were the most effective treatments for weight loss (Figure 3 A). Simultaneous comparison of all treatments in this network is shown in supplementary Table 3.

***Network 2***: This network involved three RCTs with five treatments and five pairwise comparisons (Figure 2 B). The behavior modification+exercise (LED) versus low-fat diet (LFD) was more effective [P=0.99, MD=-6.60 (-9.07, -4.13)]. In addition, the low-carbohydrate diet (LCD), Mediterranean diet (MedDiet), and Mediterranean/LCD+28 g walnuts (MEDLCD+walnuts) were more effective than LFD (Figure 3 B). Simultaneous comparison of all treatments in this network is shown in supplementary Table 4.

**Figure 4:**
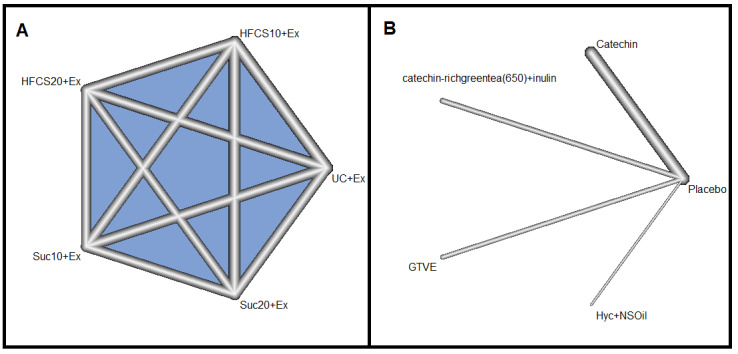
Networks three (A) and four (B) for the interventions for the treatment of obesity.

***Network 3:*** The third network involved one RCT with five treatments and 10 pairwise comparisons (Figure 4 A). The I^2^ statistic in this network was zero. Moreover, there was no statistically significant difference between treatments in this network. However, sweetener at 20% of total calories (HFCS20)+Ex with [P=0.67, MD=-3.75 (-10.24, 2.74)] achieved the highest rank (Figure 4 A). Simultaneous comparison of all treatments in this network is shown in supplementary Table 5.

**Hyc:** Hypocaloric diet; **Hyc+AVO**: Hypocaloric diet+Hass avocado daily; **Hyc+MonCam (150):** Hypocaloric diet+150 mg Monoselect Camellia; **Hyc+WPS**: Hypocaloric diet+supplementation with whey protein, 0.5 g/kg;
**VegestartComplet**: balanced energy and high protein formula diet;
**Whey+RT+Hyc**: whey protein hydrolysate+resistance exercise+hypocaloric diet; **Casein+RT+Hyc**: casein protein hydrolysate+resistance exercise+hypocaloric diet; **LED**: Energy restriction (4,186 kJ/day to 5,023 kJ/day), behavior modification+exercise; ** LFD**: low-fat diet; **LCD**: low-carbohydrate diet; **MedDiet**: Mediterranean diet; **MEDLCD+walnuts (28)**: Mediterranean/low-carbohydrate diet+28 g walnuts

**MD:** Mean difference; **CI:** confidence interval; **Hyc :** hypocaloric diet; **Hyc+MonCam :** hypocaloric diet+Monoselect Camellia; **VegestartComplet**: balanced energy and high protein formula diet; **Hyc+WPS**: hypocaloric diet+supplementation with whey protein; **Casein+RT+Hyc**: casein protein hydrolysate+resistance exercise+hypocaloric diet; **Whey+RT+Hyc**: whey protein hydrolysate+resistance exercise+hypocaloric diet; **Hyc+AVO**: Hypocaloric diet+Hass avocado daily; **LED:** energy restriction, behavior modification+exercise; **MEDLCD+walnuts :** Mediterranean/low-carbohydrate diet+28 g walnuts; **LCD**: low-carbohydrate diet; **MedDiet**: Mediterranean diet, **LFD**: low-fat diet

***Network 4***: This network involved four RCTs with five treatments and four pairwise comparisons (Figure 4 B). The I^2^ value was zero, and Catechin was significantly more effective, compared to placebo in weight loss (MD=-1.20; 95% CI: -2.21, -0.19). However, catechin-rich green tea (650)+inulin [P=0.68, MD=-1.90 (-4.00, 0.20)] had the highest treatment rank among other treatments (Figure 4 B). Simultaneous comparison of all treatments in this network is shown in supplementary Table 6.

**HFCS10+Ex**: sweetener at 10% of total calories+exercise; **HFCS20+Ex**: sweetener at 20% of total calories+exercise; **Suc10+Ex**: 10% Sucrose+exercise; **Suc20+Ex**: 20% sucrose+exercise; **UC+Ex**: eucaloric diet+exercise; **catechin- richgreentea (650)+ inulin**: catechin-rich green tea (650)+inulin; **GTVE**: green tea+vitamin E; **Hyc+NSOil**: low-calori diet with 3 g/day; **Catechin**: beverage containing 625 mg of catechins

***Network 5:*** This network consisted of three RCTs with four treatments and three pairwise comparisons (Supplementary figure 2 A). The I^2^ statistic was zero, and the very low calorie diet (VLCD) [P=1.00, MD=-4.50 (-5.31, -3.69)] and low-fat vegan diet [P=0.72, MD=-2.00 (-3.54, -0.46)] were significantly more effective than eucaloric diet (UC) (supplementary Figure 3A). The simultaneous comparison of all treatments in this network is shown in supplementary Table 7.

***Network 6:*** This network involved one RCT with four treatments and six pairwise comparisons (supplementary Figure 2 B). Normal protein diet (0.8 g/kg)+resistance exercise (NPD+RT) (P=0.80, MD=-0.90 [-2.26, 0.46]) was the most effective treatment, compared to the other treatments in this network (supplementary Figure 3B). Simultaneous comparison of all treatments in this network is shown in supplementary Table 8.

***Network 7:*** This network comprised two RCTs with four treatments and four pairwise comparisons (supplementary Figure 4 A). The Hyc+Ex (calorie restriction+exercise) was the most effective treatment in comparison with the other treatments in this network (P=0.85, MD=-4.45 [-4.72, -4.18]) (Supplementary Figure 5 A). Simultaneous comparison of all treatments in this network is shown in supplementary Table 9.

***Network 8:*** This network was a three arms RCT with three treatments and three pairwise comparisons (supplementary figure 4B). The high-soy-protein low-fat diet (SD) and SD-physical activity (PA) versus LE (lifestyle education) reduced the weight of participants significantly (P=0.75) (supplementary Figure 5B). Simultaneous comparison of all treatments in this network is shown in supplementary Table 10.

***Network 9:*** This network was formed by two RCTs with three treatments and two pairwise comparisons (supplementary Figure 6). The Hyc (calorie restriction to 1000-1200 kcal/day)+behavioral weight loss (BWL) treatment with (P=0.99, MD=-5.70 [-10.14, -1.26]) was the most effective treatment in this network (supplementary Figure 7). Simultaneous comparison of all treatments in this network is shown in supplementary Table 11.

**Figure 5:**
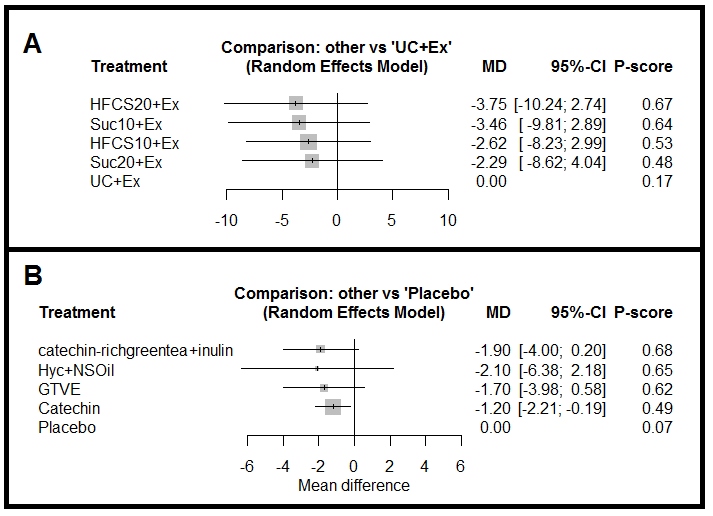
Forest plot for comparison of all treatments in network three with UC+Ex (A) and all treatments in network four with placebo (B) and related p-value for each treatment.

**MD:** Mean difference; **CI:** confidence interval; **HFCS10+Ex**: sweetener at 10% of total calories+exercise; **HFCS20+Ex**: sweetener at 20% of total calories+exercise; **Suc10+Ex**: 10% Sucrose+exercise; **Suc20+Ex**: 20% sucrose+exercise; **UC+Ex**: eucaloric diet+exercise; **GTVE**: green tea+vitamin E; **Hyc+NSOil**: low-calori diet with 3 g/day; **Catechin**: beverage containing catechin

## Discussion

 In this systematic review and NMA, the available diet therapeutics interventions to treat obesity were simultaneously compared. The treatments were ranked based on their effects on weight loss. Based on the results, the available interventions formed nine separate networks. In addition, 28 treatments in 14 RCTs were not connected to any network, and their results were reported separately. Overall, Hyc+MonCam, LED, HFCS20+Ex, catechin-rich green tea (650)+inulin, VLCD, NPD+RT, Hyc+Ex, SD, Hyc+BWL were the better treatments for weight loss in the networks of this study.

In the first network with seven treatments, Hyc+MonCam (150), VegestartComplet treatment, and Hyc+VPS versus Hyc alone were the most effective treatments. In this network, the highest p-value was related to Hyc+MonCam. The effect of MonCam in reducing the waistline is shown in a systematic review ^26^. MonCam is an oral formulation containing highly bioavailable green tea extract. Green tea plays a vital role in the metabolism of fat by reducing food intake, disturbing lipid absorption and emulsification, suppression of lipid synthesis, fat oxidation, fecal lipid excretion, and increase of the energy expenditure^27^. In addition, the safety of MonCam is proved, and there are no complications in using this product^28^.

Other treatments in this network, such as Vegestart-Complet and Hyc+supplementation with whey protein had a high p-value and were significantly more effective in weight loss than a Hyc. Based on the results of a study, the people in the energy-restricted condition lost over twice as much weight as those in the fat-restricted group^29^.

The results in the second network with five treatments showed energy restriction and LED was the most effective treatment for weight loss, compared to LFD, LCD, MedDiet, and MEDLCD+walnuts. Based on the results of the published studies, both intermittent and continuous energy restrictions were effective in weight loss^30,31^. The MEDLCD+walnuts diet was the second-best treatment in this network. According to the results of a published study, the Mediterranean/low-carbohydrate diet was more influential in the decrease of hepatic fat, compared to the low-fat diet and had more favorable health effects than visceral fat loss^32^.

The third network involved an RCT with five treatments, including HFCS10+Ex, HFCS20+Ex, Suc10+Ex, Suc20+Ex, and UC+Ex^11^. Based on our analysis, there was no statistically significant difference between the effects of these treatments on weight loss. In this meta-analysis, HFCS10+Ex, HFCS20+Ex, Suc10+Ex, Suc20+Ex versus UC+Ex were compared; although these treatments were more effective than UC+Ex, the difference was not statistically significant.

In the fourth network with five treatments, catechin-rich green tea+inulin achieved the highest rank. Previous studies had shown the effects of catechin green tea on body composition. High catechin green tea leads to the reduction of abdominal fatness among overweight and obese people^33^. On the other hand, the effect of inulin on weight loss has been shown in animal^34^ and human studies^35^.

In the fifth network with four treatments, VLCD was the first rank treatment. The VLCD versus UC reduced the weight of the participants by about 4.5 kg. Based on the results of a meta-analysis, a very-low-calorie ketogenic diet (VLCKD) was associated with a reduction in waist circumference, BMI, HbA1c, total cholesterol, triglycerides, ALT, AST, GGT, and systolic and diastolic blood pressures in people with obesity. Based on the results of the aforementioned meta-analysis, VLCKD was an effective strategy for weight loss among people with overweight and obese^36^.

Network number six was an RCT with four treatments^37^. Based on the results of the present analysis, there was no significant difference among HPD (high-protein diet), NPD (normal-protein diet), NPD+RT+resistance exercise, and HPD+RT+resistance exercise; however, the highest p-value was related to NPD+RT. These results are not consistent with a review which concluded that HPD decreases body weight^38^. Nevertheless, HPD modifies the microbiota activity and gene expression in the rectal mucosa; hence, caution should be exercised regarding the utilization of HPD^38^.

In network number seven, four treatments, including Ex, Ex+CC, Hyc+Ex, and NCDs+Ex, were compared simultaneously. In this network, Hyc+Ex and Ex+CC were significantly more effective than Ex alone. However, the highest p-value was related to Hyc+Ex. The effect of physical activity and diet on weight loss has been shown in the previous studies^39^. Results of the present study are in line with those of a meta-analysis that showed the combined diet+exercise was more effective than diet alone^40^. Physical activity increases the total energy expenditure and decreases total body fat ^41^. Based on the results of a study, physical activity and Hyc are associated with the reduction of oxidative stress in serum and pro-oxidant effect on hepatic tissue and reducing antioxidant defenses^42^.

Network number eight was a three-arm RCT with three treatments, including SD, LE, and SD-PA^43^. Based on our results, both SD and SD-PA were more effective than LE, while there was no statistically significant difference between SD and SD-PA. In this RCT, there was no significant difference between groups in terms of biochemical parameters, such as total cholesterol, HDL-cholesterol, and LDL-cholesterol. However, this network involved only one RCT with three treatments; more RCTs are needed for better inference of the effectiveness of these interventions.

The last network in this NMA involved three treatments, including LCD+BWL, LFD+BWL, and Hyc+BWL. In this network, Hyc+BWL was the most effective treatment for weight loss. The effect of hypo calorie diet is shown in the previous studies^36^. Moreover, behavioral interventions have been recommended in weight loss programs. In addition, the behavioral interventions for weight loss are associated with less weight gain after the termination of interventions^44^. Therefore, it is expected that the combination of Hyc and behavioral interventions be more useful in weight loss.

It must be mentioned that in this NMA, we had some limitations. Firstly, the included RCTs in this systematic review formed nine separate networks. Although we compared all treatments simultaneously in each network, we could not compare all treatments in a single network simultaneously. Secondly, the number of RCTs in some networks was low. This issue affected the power of networks for estimating the indirect effect sizes and, we were exposed to wide CIs in some indirect effect sizes^45^; hence, the sparse data bias might have affected our results.

Another limitation was the small number of RCTs in the networks. Consequently, due to the low power and lack of validity for statistical tests for the assessment of publication bias, we could not evaluate this bias in the present NMA^46^. Another limitation was the lack of access to the full text of some RCTs which may increase the risk of bias in the results. This issue raises the risk of publication bias; hence, in the absence of these studies, the results of NMA in a network may differ from reality.

 This study is a comprehensive research that evaluated the available diet therapeutics interventions for weight loss. The available RCTs on diet therapeutics interventions were collected in a single review, hence, we think this study may be valuable for decision making. However, it seems that more RCTs are needed for a better understanding of the effectiveness of anti-obesity treatments.

## Conclusion

 Based on the results of this NMA, it seems that Hyc+MonCam, LED, HFCS20+Ex, catechin-rich green tea+inulin, VLCD, NPD+RT, Hyc+Ex, SD, and Hyc+BWL are the better treatment options for weight loss in patients with overweight and obesity.

## Acknowledgments

 This study was derived from a thesis submitted in partial fulfillment of the requirement for the degree of M.Sc. in Epidemiology. The authors would like to thank the Health Sciences Research Center and the Research and Technology Deputy of the Hamadan University of Medical Sciences for supporting this study.

## Conflict of interests

 The authors declare that they have no competing interests.

## Funding

 This work was supported by the Hamadan University of Medical Sciences [9810177955].

## Highlights

- This network meta-analysis was conducted for the simultaneous comparison of diet therapeutic interventions for obesity.
- There were nine separated networks for available diet therapeutic interventions for obesity.
- Treatments in each network were ranked based on their effectiveness on weight loss.
